# Mayo adhesive probability score predicts perioperative outcomes of laparoscopic adrenalectomy: a systematic review and meta-analysis

**DOI:** 10.3389/fonc.2026.1753320

**Published:** 2026-05-13

**Authors:** Zhiqiang Zeng, Yang He, Peng Ji, Lunhong Zou, Tao Li, Yubo Zhou, Tao Zhou, Huan Zhao, Xionglin Hu, Wangbing Chen, Wubin Chen

**Affiliations:** 1 Department of Urology, Santai People’s Hospital (Santai Hospital Affiliated to North Sichuan Medical College), Mianyang, Sichuan, China; 2 North Sichuan Medical College (University), Nanchong, Sichuan, China

**Keywords:** adhesive periadrenal fat, laparoscopic adrenalectomy, mayo adhesive probability score, meta-analysis, perioperative outcomes

## Abstract

**Objective:**

This meta-analysis aims to systematically evaluate the association between the Mayo Adhesive Probability (MAP) score and perioperative outcomes of laparoscopic adrenalectomy (LA) for benign adrenal tumors, and to explore the value of the MAP score in preoperative risk stratification.

**Method:**

A comprehensive literature search was conducted in Embase, PubMed, Cochrane Library, and Web of Science from inception to June 1, 2025, to identify studies investigating the correlation between MAP score and LA outcomes. Eligible studies were selected based on predefined criteria, and data extraction was performed independently by two reviewers. The primary outcomes included operative time (OT), estimated blood loss (EBL), length of stay (LOS), and postoperative complications. In addition, baseline and clinicopathological characteristics, including body mass index (BMI), gender distribution, hormonal activity, diabetes mellitus history, and hypertension history, were compared between the study-defined higher MAP and lower MAP groups. Meta-analysis was performed using Stata 16.0.

**Results:**

A total of 5 retrospective studies involving 578 patients were included. A meta-analysis showed that, compared with the low MAP score group, the high MAP score group had significantly longer OT, higher EBL, and a higher risk of complications. No significant difference in LOS was observed between the two groups. Additionally, the high MAP score group had a higher BMI, a higher proportion of male patients, and more frequent hormonal activity. There were no significant differences in the history of diabetes mellitus or hypertension between the groups.

**Conclusions:**

Higher MAP scores were associated with longer operative time, greater estimated blood loss, and a higher complication rate in laparoscopic adrenalectomy, while no significant difference in length of stay was observed. The MAP score may be a useful adjunct for preoperative risk assessment, although prospective validation is still needed.

## 1 Introduction

Laparoscopic adrenalectomy (LA) has gradually become the standard surgical procedure for treating benign small adrenal tumors (1, 2), offering advantages over open surgery such as reduced invasiveness, shorter recovery, and fewer complications (3). Despite its efficacy, LA faces challenges related to anatomical complexity and patient-specific factors, such as the anatomic localization of the adrenals, dissection of periadrenal fat, ligation of the main adrenal vein, and separation of the posterior adrenal planes from the liver or spleen (4). Research indicates that obesity, as measured by body mass index (BMI), is associated with poorer surgical outcomes, including prolonged operative time and increased blood loss (5–7). However, some research reports contradictory results (8, 9), highlighting the need for more precise preoperative risk-stratification tools.

The Mayo Adhesive Probability (MAP) score, initially developed to predict adherent perinephric fat (APF) in partial nephrectomy (10), may emerge as a promising imaging-based metric for adrenal surgery. This score integrates two radiological features on preoperative computed tomography (CT): Perinephric fat thickness and Perinephric fat stranding. Given the adrenal gland’s anatomical position within perinephric fat, the MAP score potentially correlates with dissection challenges in LA (11). Recent studies confirm the score’s utility in forecasting perioperative outcomes. Specifically, higher MAP scores are significantly associated with prolonged operative time (OT), increased estimated blood loss (EBL), and reduced hemoglobin levels in retroperitoneal LA (11). These trends are observed uniformly across total and partial adrenalectomy techniques, in which elevated MAP scores independently predict complications (12).

Although previous systematic reviews and meta-analyses have supported the utility of the MAP score in predicting technical difficulty during partial nephrectomy (13, 14), evidence regarding its role in laparoscopic adrenalectomy remains limited. Therefore, we performed a systematic review and meta-analysis to evaluate the association between MAP score and perioperative outcomes in LA.

## 2 Methods

### 2.1 Literature search

We conducted a thorough systematic review and a comprehensive meta-analysis, primarily on key outcomes, in alignment with the PRISMA criteria (15). Adhering to the AMSTAR guidelines ensured the quality of the assessment. Our systematic review is registered on PROSPERO.

Two researchers independently executed literature searches and screening across four databases (Embase, PubMed, Cochrane Library, Web of Science) from inception until June 1, 2025. Search strategies incorporated key terms and synonyms related to the MAP score and laparoscopic adrenalectomy, including both transperitoneal and retroperitoneal approaches: (“Mayo adhesive probability score” OR MAP) AND (“laparoscopic adrenalectomy” OR “retroperitoneal adrenalectomy” OR “retroperitoneoscopic adrenalectomy” OR “posterior retroperitoneal adrenalectomy” OR “transperitoneal adrenalectomy”). Article eligibility was determined through independent title/abstract assessment by dual investigators; unresolved discrepancies were adjudicated by a third expert. Manual examination of relevant study bibliographies supplemented the search.

### 2.2 Eligibility criteria

Reports qualified for inclusion in this systematic review based on these criteria: (1) Studies involving patients undergoing LA; (2) Evaluation of posterior perinephric fat thickness and perinephric fat stranding employing the MAP scoring system; (3) Contained a minimum of one perioperative outcome, such as OT, length of stay (LOS), EBL, and postoperative complications. Exclusion criteria covered: (1) Inability to extract usable data; (2) Study comprising editorials, conference materials, or expert opinions; (3) Duplicate investigations reporting identical findings within studied populations; (4) Studies utilizing subjects other than humans; (5) Omission of the MAP scoring system for LA.

### 2.3 Data extraction

Two separate reviewers selected relevant articles and gathered information using a data collection table. The collected information comprised various details: author, publication date, document type, sample capacity, age, tumor localization, approach method, BMI, tumor size, operative methods, OT, LOS, EBL, and post-operative complications.

### 2.4 Study quality assessment

Retrospective investigations were evaluated using the Newcastle-Ottawa Scale (NOS) 2. NOS scores range from 0–9, with≥7 indicating high-quality methodology.

### 2.5 Risk of bias assessment

Two reviewers independently assessed potential biases in selected studies using the ROBINS-I tool, specifically designed for non-randomized research. This tool assesses bias within seven areas: confounding bias, selection bias, intervention measurement classification bias, bias due to deviations from the intended intervention, bias due to missing data, outcome measurement bias, and reported outcome selection bias2 (16).

### 2.6 Data analysis

Data analysis was conducted using Stata 16.0 (StataCorp LLC, Address: 4905 Lakeway Dr, College Station, TX 77845). To synthesize outcomes across all included trials, we used log OR (Odds Ratio) and WMD (Weighted Mean Difference) (17). Statistical significance was defined as P<0.05. Heterogeneity among studies was evaluated using Chi-square and Q tests; thresholds of I² > 50% or P < 0.10 indicated significant between-study heterogeneity, prompting selection of a random-effects model. The MAP score ranged from 0 to 5 in the included studies. Because the original studies used different cutoffs to dichotomize MAP scores, we did not impose a uniform threshold across all studies. Instead, for the overall pooled analysis, patients were compared as the study-defined higher-MAP group versus the study-defined lower-MAP group, using each study’s original categorization. To explore whether different thresholds affected the pooled estimates, subgroup analyses were performed according to the cutoff used in the original study. In studies using MAP ≥2 as the threshold, the lower-MAP group was defined as MAP 0–1 and the higher-MAP group as MAP 2–5. In studies using MAP ≥3 as the threshold, the lower-MAP group was defined as MAP 0–2 and the higher-MAP group as MAP 3–5.

## 3 Results

### 3.1 Description of study

**Figure 1:**
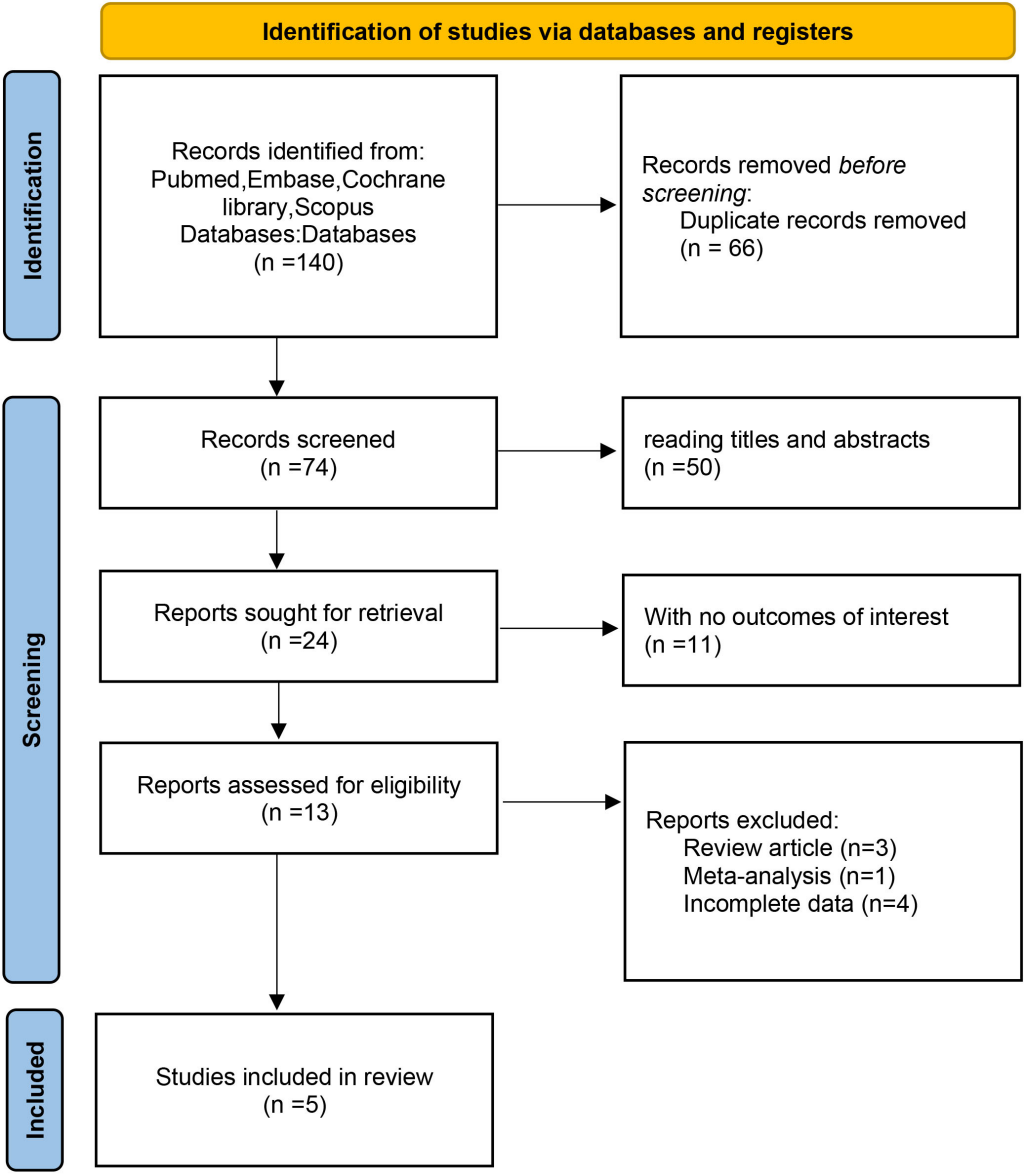
Flow diagram of the studies selection process.

The authors searched 140 records from four databases. 66 duplicate studies were removed; After title and abstract screening, 50 records were excluded because they did not meet the predefined eligibility criteria; 11 studies had no outcomes of interest; 3 systematic reviews, 1 meta-analysis, and 4 had incomplete data. A total of 5 studies involving 578 patients were included in this meta-analysis. In addition, the sample size ranged from 46 to 186. All 5 studies were retrospective studies (12, 18–21). The screening process is shown in **Figure 1**, and the baseline characteristics of the included studies are shown in **Table 1**. 5 publications were published from 2022 to 2025.

**Table 1:** Baseline data for studies included in the meta-analysis.

Author	Year	Type	Sample (n)	Age	Operation	Tumor Size(cm)	Center	BMI^a^(kg/m2)	Approach method
Baylan, B.	2025	Retrospective	46	53.4	Laparoscopic	5	Single-Center	24.1	Intraperitoneal/Retroperitoneal
Miyamoto, T.	2024	Retrospective	103	58	Laparoscopic	3	Single-Center	24	Intraperitoneal/Retroperitoneal
Tuncel, A.	2024	Retrospective	139	49.4	Laparoscopic	4.2	Single-Center	30.02	Intraperitoneal
Yuan, Y.	2022	Retrospective	104	48.1	Laparoscopic	2.04	Single-Center	24.3	Retroperitoneal
Chen, W.	2022	Retrospective	186	37.3	Laparoscopic	2.1	Single-Center	25	Retroperitoneal

BMI^a^ = body mass index.

### 3.2 Quality assessment

The quality of the cohort studies was evaluated using the modified Newcastle-Ottawa Scale, and the NOS scores ranged from 6 to 7. 5 studies were included in the assessment, all with a score of 6 or more in **Table 2**.

**Table 2:** Quality score of included studies based on the NOS scale.

Study	Selection			Comparability			Exposure			Total stars
	aREC	bSNEC	cAE	dDO	eSC	fAF	gAO	hFU	iAFU	
Baylan, B.	1	1	1	1		1	1	1		7
Miyamoto, T.	1	1	1		1		1		1	6
Tuncel, A.	1	1	1	1				1	1	6
Yuan, Y.	1	1	1	1	1	1	1			7
Chen, W.	1		1	1	1	1	1	1		7

a REC, representativeness of the cohort.

b SNEC, selection of the none posed cohort.

c AE, ascertainment of exposure.

d DO, demonstration that outcome of interest was not present at start of study.

e SC, study controls most important factors.

f AF, study controls for other important factors.

g AO, assessment of outcome.

h FU, follow-up long enough for outcomes to occur.

i AFU, adequacy of follow-up of cohort (≥ 80%).

### 3.3 Operation time

**Figure 2:**
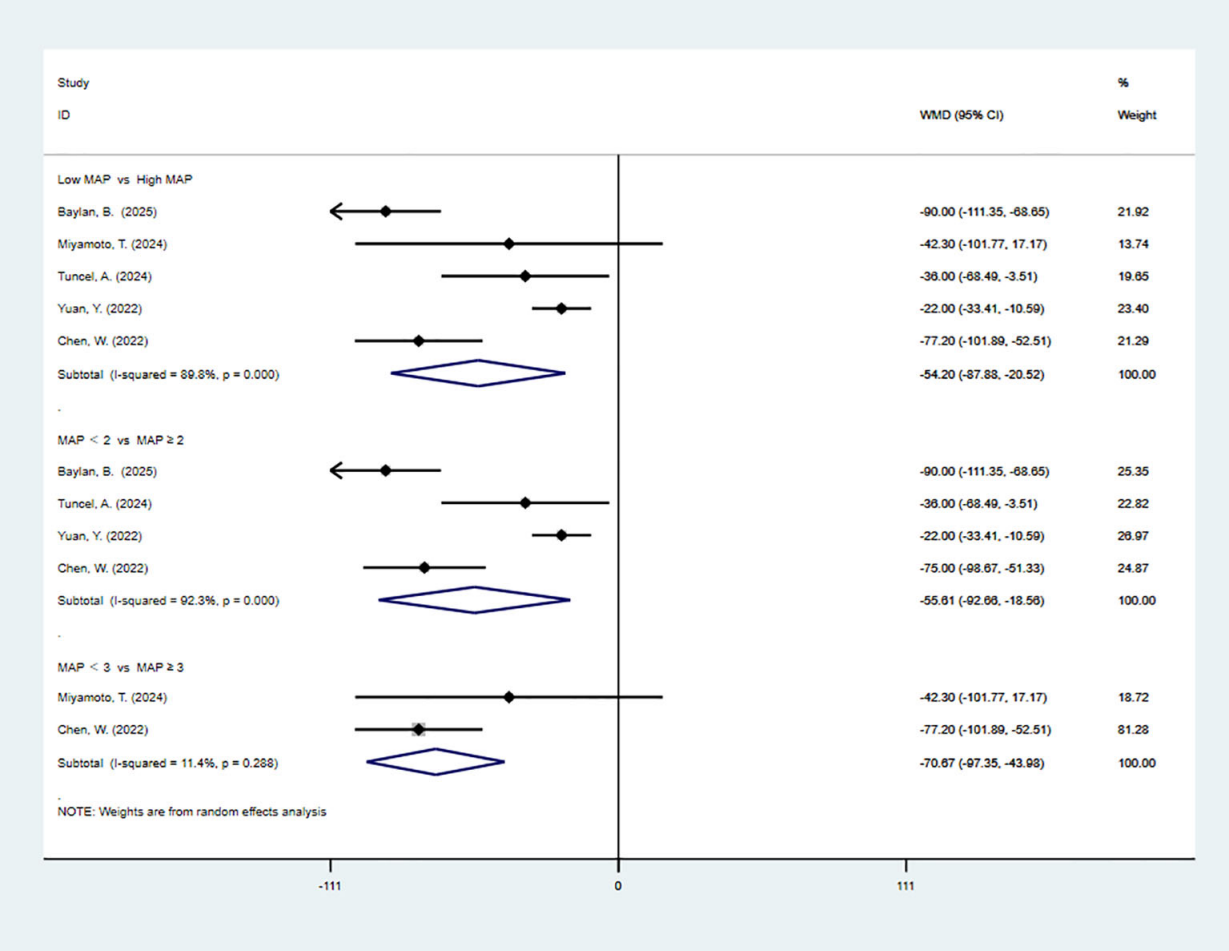
Forest plot and meta-analysis of OT.

5 studies reported OT. The pooled meta-analysis demonstrated a significant difference between the high-MAP-score-group and the low-MAP-score-group (WMD = -34.74, 95% CI [-56.92, -12.56], P < 0.05). 4 articles are divided by more than or equal to 2. The pooled meta-analysis demonstrated a significant difference between the high-MAP-score-group and the low-MAP-score-group (WMD = -34.26, 95% CI [-58.47, -10.06], P < 0.05). 2 articles are divided by more than or equal to 3. The pooled meta-analysis demonstrated a significant difference between the high-MAP-score-group and the low-MAP-score-group (WMD = -34.34, 95% CI [-45.21, -23.47], P < 0.05) (**Figure 2**).

### 3.4 Length of stay

**Figure 3:**
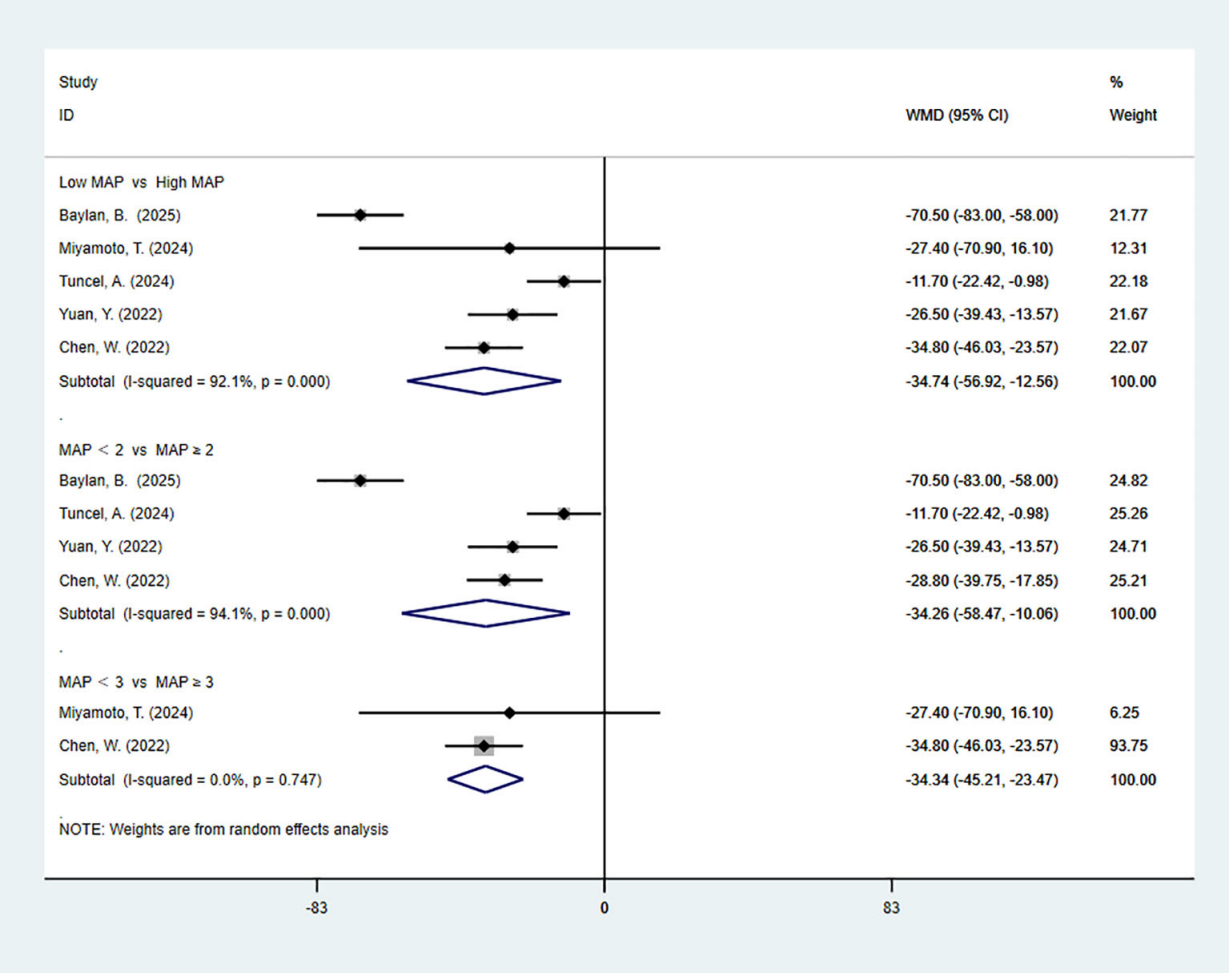
Forest plot and meta-analysis of LOS.

4 studies reported LOS. The pooled meta-analysis demonstrated no significant difference between the high-MAP-score-group and the low-MAP-score-group (WMD = -0.95, 95% CI [-1.92, 0.02], P > 0.05). 3 articles are divided by more than or equal to 2. The pooled meta-analysis demonstrated no significant difference between the high-MAP-score-group and the low-MAP-score-group (WMD = -0.90, 95% CI [-2.25, 0.45], P > 0.05) (**Figure 3**).

### 3.5 Estimated blood loss

**Figure 4:**
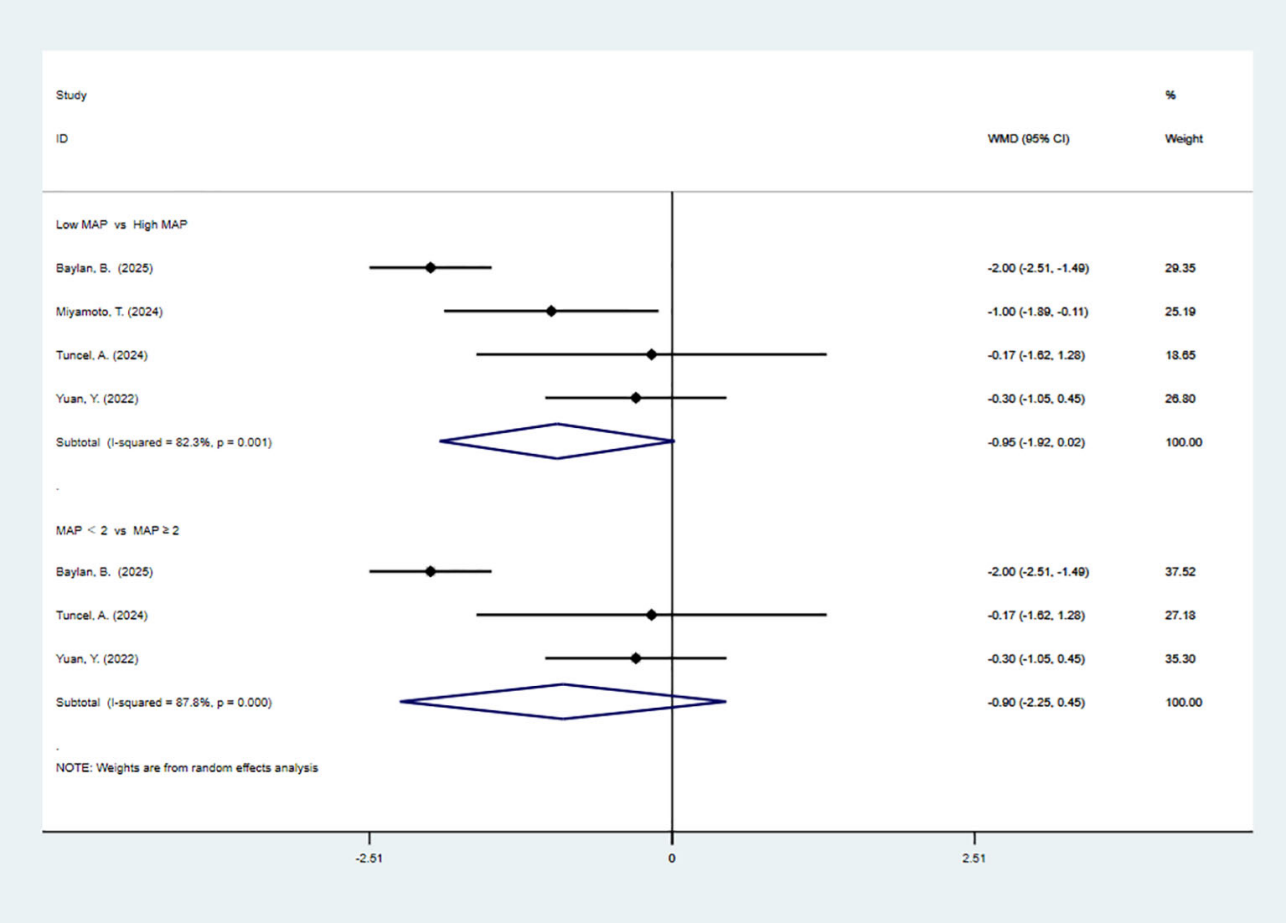
Forest plot and meta-analysis of EBL.

5 studies reported EBL. The pooled meta-analysis demonstrated a significant difference between the high-MAP-score-group and the low-MAP-score-group (WMD = -54.20, 95% CI [-87.88, -20.52], P < 0.05). 4 articles are divided by more than or equal to 2. The pooled meta-analysis demonstrated a significant difference between the high-MAP-score-group and the low-MAP-score-group (WMD = -55.61, 95% CI [-92.66, -18.56], P < 0.05). 2 articles are divided by more than or equal to 3. The pooled meta-analysis demonstrated significant difference between the high-MAP-score-group and the low-MAP-score-group (WMD = -70.67, 95% CI [-97.35, -43.98], P < 0.05) (**Figure 4**).

### 3.6 Complications

**Figure 5:**
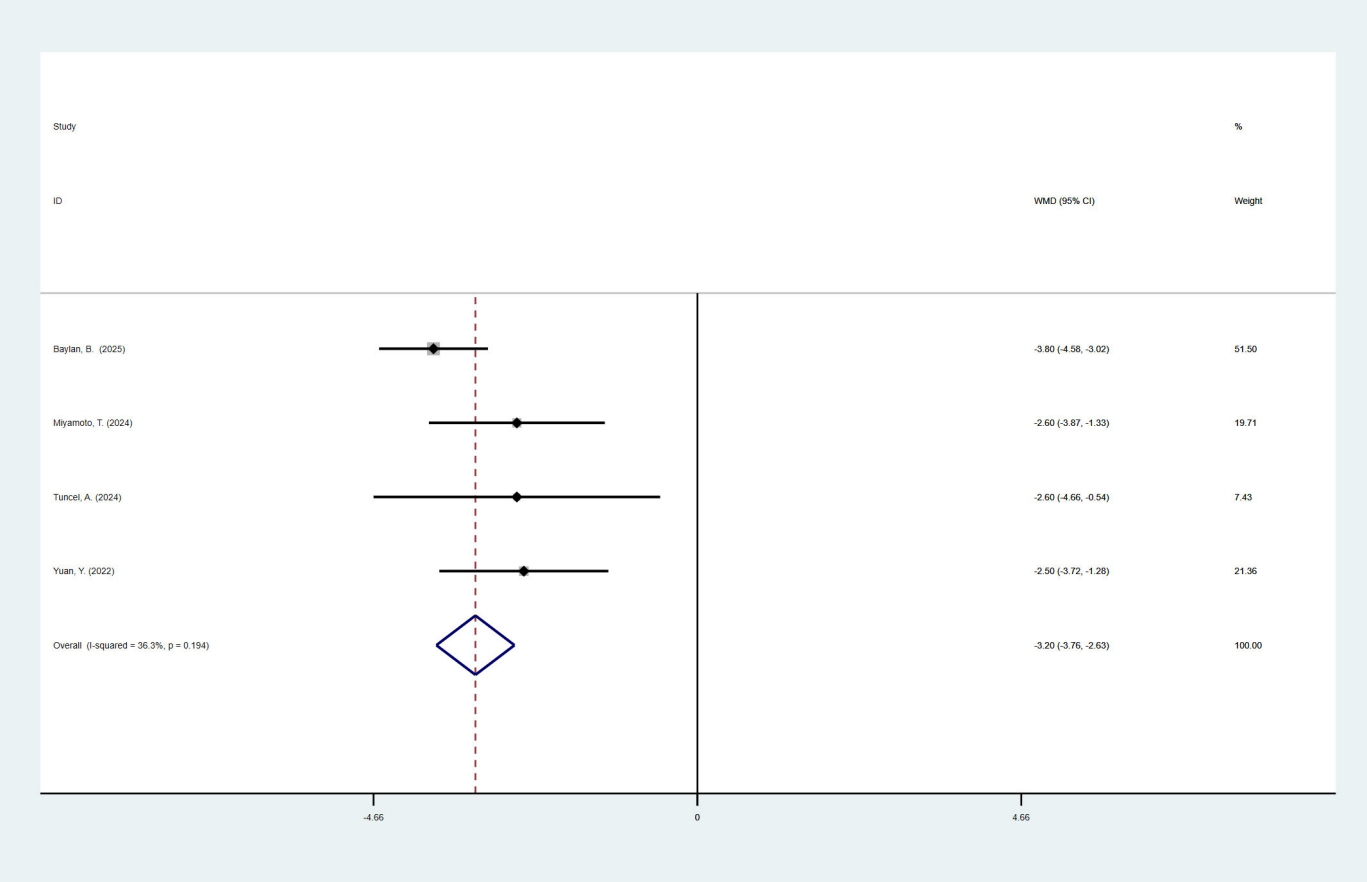
Forest plot and meta-analysis of complications.

5 studies reported complications. The pooled meta-analysis demonstrated a significant difference between the high-MAP-score-group and the low-MAP-score-group (OR = 0.34, 95% CI [0.19, 0.64], P < 0.05). 3 articles are divided by more than or equal to 2. The pooled meta-analysis demonstrated a significant difference between the high-MAP-score-group and the low-MAP-score-group (OR = 0.26, 95% CI [0.12, 0.58], P < 0.05). 2 articles are divided by more than or equal to 3. The pooled meta-analysis demonstrated no significant difference between the high-MAP-score-group and the low-MAP-score-group (OR = 0.57, 95% CI [0.20, 1.63], P > 0.05) (**Figure 5**).

### 3.7 Body mass index

**Figure 6:**
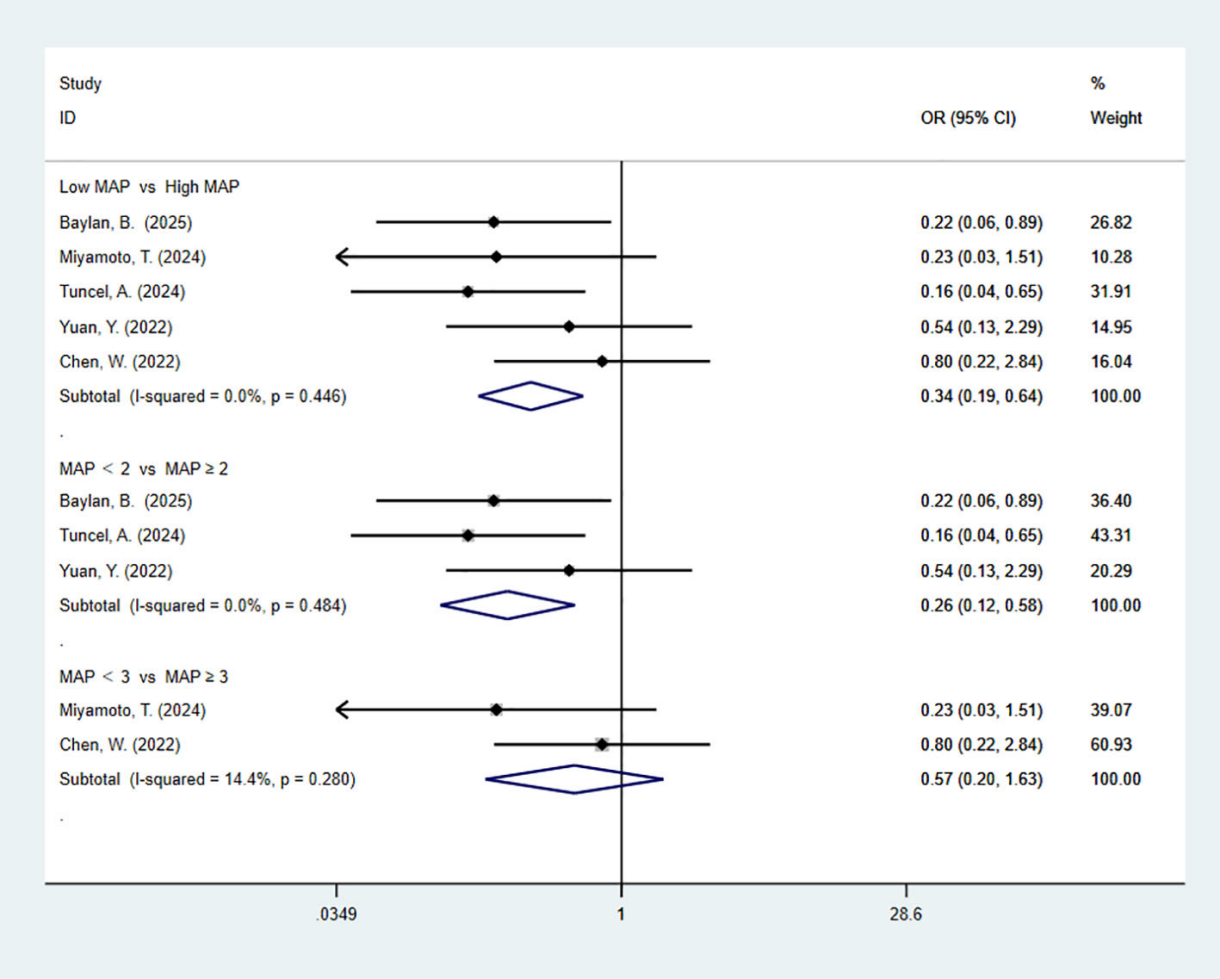
Forest plot and meta-analysis of BMI.

4 studies reported BMI. The pooled meta-analysis demonstrated significant difference between the high-MAP-score-group and the low-MAP-score-group (WMD = -3.20, 95% CI [-3.76, -2.63], P < 0.05) (**Figure 6**).

### 3.8 Baseline and clinicopathological characteristics

**Figure 7:**
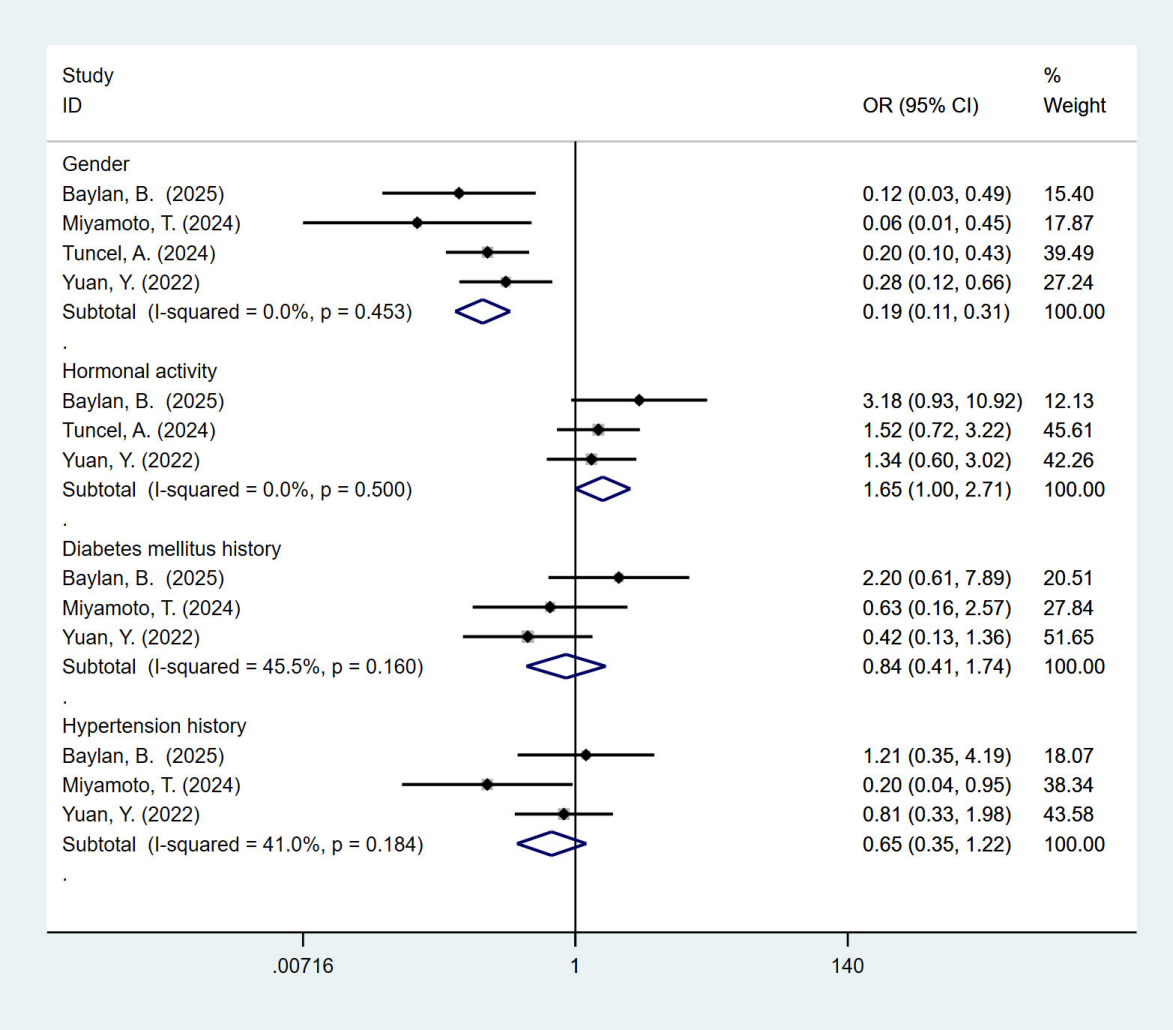
Forest plot and meta-analysis of baseline and clinicopathological characteristics, including sex distribution, hormonal activity, diabetes mellitus history, and hypertension history.

4 studies reported gender. The pooled meta-analysis demonstrated a significant difference between the high-MAP-score-group and the low-MAP-score-group (OR = 0.19, 95% CI [0.11, 0.31], P < 0.05). 3 studies reported hormonal activity. The pooled meta-analysis demonstrated a significant difference between the high-MAP-score-group and the low-MAP-score-group (OR = 1.65, 95% CI [1.00, 2.71], P < 0.05). 3 studies reported diabetes mellitus history. The pooled meta-analysis demonstrated no significant difference between the high-MAP-score-group and the low-MAP-score-group (OR = 0.84, 95% CI [0.41, 1.74], P > 0.05). 3 studies reported a history of hypertension. The pooled meta-analysis demonstrated no significant difference between the high-MAP-score-group and the low-MAP-score-group (OR = 0.65, 95% CI [0.35, 1.22], P > 0.05) (**Figure 7**).

## 4 Sensitivity analysis

We used sensitivity analyses to assess robustness and identify sources of heterogeneity for each outcome measure. The findings revealed that the source of heterogeneity is stable.

## 5 Discussion

This meta-analysis synthesized key findings from relevant studies: (1) The MAP score, which quantifies posterior perinephric fat thickness and stranding, is an independent predictor of perioperative outcomes in LA, with higher scores correlating with longer OT, higher EBL, and more complications. (2) several baseline and clinicopathological characteristics, including male sex, higher BMI, and hormonal activity, were more frequently observed in higher-MAP groups002E.

Adrenal tumors are found in 1.4% to 7% of adults undergoing abdominal imaging, with a tenfold increase in incidence over the past 30 years (22–24). Benign adrenal tumors, mainly adrenocortical adenomas, account for 70% to 95% of newly diagnosed cases of adrenal tumors (25, 26). These tumors are categorized into functional and non-functional subtypes: functional tumors often present with distinct clinical manifestations such as hypertension, electrolyte disturbances, or metabolic disorders, while non-functional tumors are frequently asymptomatic (27, 28). Diagnostically, the workup relies on a combination of endocrine evaluations and imaging modalities to characterize tumor function and anatomy (29–31). Surgical resection remains the cornerstone of treatment for symptomatic or potentially malignant tumors, with minimally invasive techniques now prevailing over open surgery due to reduced perioperative morbidity (32–34).

As surgical methods advance in maturity, the preoperative evaluation of difficulty and surgical risk gains growing importance. Consequently, systematically assessing complexity and choosing the optimal surgical approach remain key concerns for surgeons. While renal surgical assessment systems have progressively developed, those for the adrenal gland remain inadequate (35–37). Past scoring system frequently overlooked the anatomical factor (38). APF complicates the stripping of perirenal fat from the kidney, leading to obscured surgical field exposure and difficulty differentiating fat from healthy tissue, thereby complicating tumor separation. It also elevates the risk of injury to the peritoneal membrane, vasculature, and even the kidney, increasing operative challenge (39). Multiple studies confirm that APF adversely affects surgical outcomes (40, 41). The MAP scoring system, incorporating posterior perinephric fat thickness and stranding, predicts APF occurrence. Mounting evidence supports the MAP score as a reliable, accurate indicator of APF, and also for predicting prolonged operative times, extended dissection periods, and greater estimated blood loss (10, 42, 43). Anatomically, adrenal glands sit superior to the kidneys and are enveloped by perirenal fat in all individuals, irrespective of obesity. Given that LA also necessitates traversing this surrounding renal environment and dissecting perinephric fat, APF likely impacts LA procedures (44). Therefore, owing to APF characteristics like thickness and stickiness, the MAP score should also find application in preliminarily assessing LA difficulty.

High MAP scores are consistently associated with longer operative times. This is primarily due to the anatomical challenges posed by APF: High MAP scores reflect increased posterior perinephric fat thickness and severe fat stranding (45). This fibrous fat binds tightly to the renal capsule and adrenal gland, blurring the avascular planes critical for safe dissection. Surgeons must spend additional time carefully separating these adhesions to avoid injury to the kidney, adrenal vein, or surrounding vasculature. Infiltrative fat stranding reduces intraoperative visibility, particularly in the retroperitoneal space. This necessitates slower, more deliberate dissection to identify key structures, further prolonging surgery. A retrospective study by Burhan Baylan et al. also supports this view that the MAP score is a key predictor of OT (12). EBL is significantly higher in high MAP groups, APF in high MAP cases is often associated with chronic inflammation and angiogenesis. MIYAMOTO et al. ‘s research confirmed that the high MAP group showed significantly higher expressions of VEGF and CD204 in APF tissues than the low MAP group (p=0.020, p=0.015) (19). This creates a hyper vascular, fragile tissue environment where even minor dissection can damage small vessels, leading to increased bleeding. The disorganized fibrous tissue in high MAP fat makes it harder to achieve precise hemostasis with clips or cautery (46). Bleeding from small vessels in the fat bed may persist longer, contributing to higher EBL. Some studies have reported that there was no statistically significant difference in the overall complication rate between the two groups (12, 18–21). However, the meta-analysis of this study found that the high MAP group had a higher risk of complications. The need for aggressive dissection in adherent fat increases the risk of iatrogenic injury. These injuries may manifest as hematomas or delayed bleeding. High MAP fat’s pro-inflammatory state, linked to CD204+ macrophage infiltration, may impair wound healing, increasing the risk of infections (19). It is worth noting that MIYAMOTO et al. (19) and Yuan et al. (21) conducted a retrospective analysis of 103 and 104 patients, respectively, and the results showed that no major complications occurred in both high-risk and low-risk groups of MAP, which may be related to the increasing maturity of laparoscopic technology. Further studies with larger samples are needed to explore the relationship between MAP and major complications.

Male patients are overrepresented in high MAP groups. Previous studies have shown that men are more likely to have APF than women (47, 48). These differences are due to the distribution of body fat between the sexes. Men typically accumulate more visceral fat compared to women, who have more subcutaneous fat (49, 50). Androgens may promote adipocyte dysfunction and pro-inflammatory cytokine release in perinephric fat, exacerbating fat stranding and adhesion. Adrenocortical hormones can affect the dysfunction of adipocytes and the release of pro-inflammatory cytokines in perirenal fat, and aggravate the adhesion and retention of fat (51, 52). Obesity increases metanephric fat thickness, which indirectly leads to an increase in MAP score. LOS data was provided by four studies. Heterogeneity testing indicated significant heterogeneity across studies (P < 0.10), which may be associated with inter-regional variations in the allocation of medical resources. The meta-analysis showed no significant difference between two group. Even if high MAP cases have longer surgeries, this is rarely prolonged enough to delay discharge, as the primary drivers of LOS, such as major complications, are relatively rare.

Recent studies suggest that fat-related surgical difficulty in laparoscopic adrenalectomy is influenced not only by fat quantity or thickness but also by its adhesive properties (7, 40, 53). In the current literature, periadrenal fat adhesion can be understood from both intraoperative and preoperative perspectives. Olcucuoglu et al. (46) described adherent periadrenal fat (APAF) as an intraoperative finding based on the difficulty of dissecting the adrenal gland from the surrounding fat tissue, and reported that approximately one-third of patients had APAF; this condition primarily prolonged operative time, without a significant effect on complication rates or hospital stay. Their study also identified diabetes mellitus and adrenal-renal fat density as independent predictors of APAF. More recently, Hu et al. (54) proposed an imaging-oriented description of APAF, emphasizing image-dense periadrenal stranding on preoperative CT as a marker associated with increased surgical difficulty. Therefore, rather than viewing these concepts as mutually exclusive, APAF may be more appropriately interpreted as a clinically relevant adhesion-related phenomenon that includes both the intraoperative manifestation of difficult fat dissection and its preoperative imaging correlates. In this context, the MAP score, as a CT-based scoring system reflecting retroperitoneal fat thickness and fat infiltration, together with other CT-derived fat characteristics, may serve as practical and readily available tools for preoperative risk stratification and for anticipating technically challenging adrenal dissection. Accordingly, MAP should be regarded as a useful preoperative screening tool that complements the operative concept of APAF and may provide a more comprehensive basis for preoperative evaluation in laparoscopic adrenalectomy. Nevertheless, further prospective studies are still needed to clarify the predictive accuracy of MAP for APAF in this setting.

From a clinical perspective, the MAP score may provide useful preoperative information for risk stratification in laparoscopic adrenalectomy. Because it is based on routine preoperative CT findings, it can be obtained noninvasively and without additional cost. A higher MAP score may alert surgeons to the possibility of more difficult dissection, longer operative time, increased blood loss, and a higher risk of complications. This information may help with operative planning, patient counseling, preparation for technically complex cases, and appropriate allocation of surgical expertise and perioperative resources. However, the MAP score should be considered a complementary tool rather than a standalone determinant of surgical decision-making.

This analysis is not without limitations. First, the included studies are all retrospective in design, which may introduce potential selection bias. Second, the MAP scoring system has inherent variability. The measurement of perinephric fat thickness and the grading of fat stranding both depend on the subjective interpretation of radiologists. Third, the included studies used different thresholds to dichotomize MAP scores, most commonly MAP ≥2 and MAP ≥3, which may have contributed to between-study heterogeneity and limited direct comparability across studies. Fourth, the sources of publications are restricted. We did not have access to unpublished research, which may inevitably result in publication bias. Fifth, the limited number of included studies and the inconsistent reporting of important operative variables, including surgical approach and extent of resection, prevented further subgroup analyses. These factors may have contributed to between-study heterogeneity and should be considered in future prospective studies with standardized reporting. Finally, long-term outcomes related to MAP scores remain insufficiently studied. Existing data primarily focus on short-term perioperative outcomes.

## 6 Conclusion

In this meta-analysis of retrospective studies, higher study-defined MAP groups were associated with longer operative time, greater estimated blood loss, and a higher overall complication rate during laparoscopic adrenalectomy for benign adrenal tumors, whereas length of stay did not differ significantly between groups. These findings suggest that the MAP score may be a useful adjunct for preoperative risk stratification; however, the current evidence is limited by the retrospective design of the included studies, the small number of available studies, and variation in MAP cutoffs across studies. Larger prospective studies with standardized methodology are needed to further define its clinical value.
